# Comparative STAT3-Regulated Gene Expression Profile in Renal Cell Carcinoma Subtypes

**DOI:** 10.3389/fonc.2019.00072

**Published:** 2019-02-26

**Authors:** Rebekah L. Robinson, Ashok Sharma, Shan Bai, Saleh Heneidi, Tae Jin Lee, Sai Karthik Kodeboyina, Nikhil Patel, Shruti Sharma

**Affiliations:** ^1^Center for Biotechnology and Genomic Medicine, Medical College of Georgia, Augusta University, Augusta, GA, United States; ^2^Department of Population Health Sciences, Medical College of Georgia, Augusta University, Augusta, GA, United States; ^3^Department of Pathology, Medical College of Georgia, Augusta University, Augusta, GA, United States; ^4^Department of Ophthalmology, Medical College of Georgia, Augusta University, Augusta, GA, United States

**Keywords:** STAT3, gene expression, TCGA, RNA-seq, renal cell carcinoma

## Abstract

Renal cell carcinomas (RCC) are heterogeneous and can be further classified into three major subtypes including clear cell, papillary and chromophobe. Signal transducer and activator of transcription 3 (STAT3) is commonly hyperactive in many cancers and is associated with cancer cell proliferation, invasion, migration, and angiogenesis. In renal cell carcinoma, increased STAT3 activation is associated with increased metastasis and worse survival outcomes, but clinical trials targeting the STAT3 signaling pathway have shown varying levels of success in different RCC subtypes. Using RNA-seq data from The Cancer Genome Atlas (TCGA), we compared expression of 32 STAT3 regulated genes in 3 RCC subtypes. Our results indicate that STAT3 activation plays the most significant role in clear cell RCC relative to the other subtypes, as half of the evaluated genes were upregulated in this subtype. *MMP9, BIRC5, and BCL2* were upregulated and *FOS* was downregulated in all three subtypes. Several genes including *VEGFA, VIM, MYC, ITGB4, ICAM1, MMP1, CCND1, STMN1, TWIST1*, and *PIM2* had variable expression in RCC subtypes and are potential therapeutic targets for personalized medicine.

## Introduction

Renal cancer is among the top ten most common cancer types globally, and 85% of renal cancers are classified as renal cell carcinomas (RCC) (1, 2). RCC tumors are heterogeneous and can be further classified into subtypes, the most common of which are clear cell (KIRC), papillary (KIRP), and chromophobe (KICH) carcinoma. Earlier studies have shown that Signal transducer and activator of transcription 3 (STAT3) signaling plays an important role in the growth of renal cancers, and increased STAT3 activation has been associated with progression of pathological stages and worse overall survival (3–5). STAT3 is a transcription factor involved in many physiological processes including cell growth, proliferation, inflammation, and apoptosis (6–9). STAT3 is activated by several cytokines and growth factors that signal through glycoprotein 130 (gp130). In response to these signals, members of the Janus-activated kinase (JAK) family phosphorylate STAT3 at Tyr^705^, which dimerizes STAT3 and translocates it to the nucleus to activate transcription (1, 7). Under normal physiological conditions STAT3 activation is tightly regulated, but in cancer an increase in extracellular signaling or the development of constitutive activity results in the aberrant expression of STAT3 regulated genes (7, 10–12). Current scientific evidence indicates that persistently activated STAT3 plays an important role in tumor onset and progression via mechanisms involving proliferation, invasion, and migration (10, 13, 14). Additionally, it can also promote cancer stem cell self-renewal and differentiation by altering gene expression through epithelial-mesenchymal transition (EMT) phenotypes in cancer cells and by regulating the tumor microenvironment (10, 15). It has also been shown to cooperate with hypoxia-inducible factor 1-alpha (HIF1A) to induce VEGF expression, thus promoting tumor angiogenesis (16).

**Figure 1:**
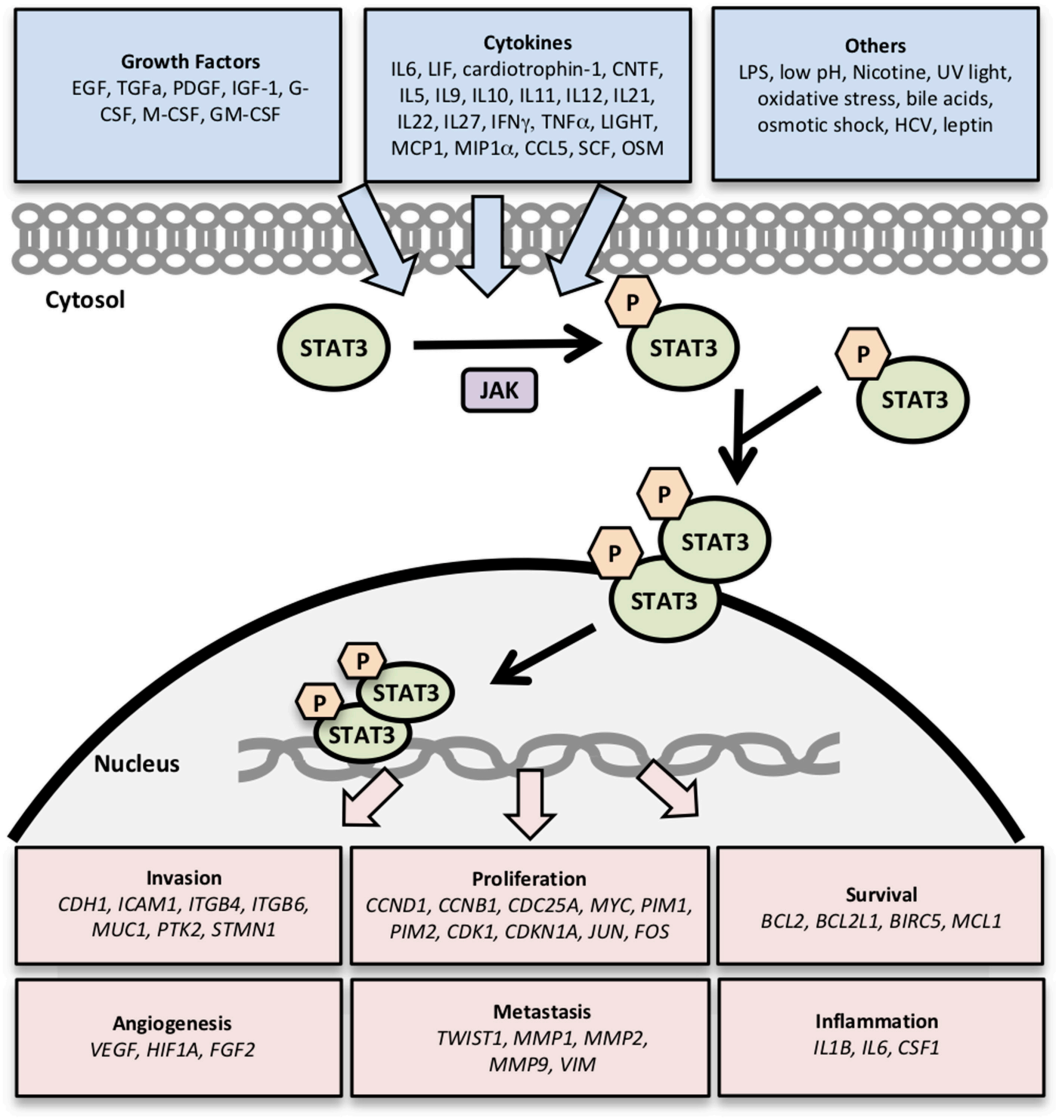
Genes regulated by STAT3 signaling. Activation of STAT3 signaling by growth factors, cytokines, and other stimuli results in the transcription of genes related to cancer cell survival, proliferation, invasion, angiogenesis, metastasis, and inflammation.

In this study, we compare and contrast the expression of clinically significant genes involved in the STAT3 pathway in different renal cancer subtypes by analyzing datasets from The Cancer Genome Atlas (TCGA). We explored the most well–characterized 32 STAT3-regulated genes involved in cancer cell invasion (*CDH1, ICAM1, ITGB4, ITGB6, MUC1, PTK2, STMN1*) (8, 12, 17–19); cell proliferation (*CCND1, CCNB1, CDC25A, MYC, PIM1, PIM2, CDK1, CDKN1A, JUN, FOS*) (8, 20–22); cell survival (*BCL2, BCL2L1, BIRC5, MCL1*) (23); angiogenesis (*VEGF, HIF1A, FGF2*) (8, 24); metastasis (*TWIST1, MMP-1*,−2,−9, *VIM*) (8, 12); and inflammation (*IL1B, IL6, CSF1*) (8, 25) (Figure 1). Then we examined the expression levels of these 32 STAT3-regulated genes in clear cell, papillary, and chromophobe RCC using the TCGA gene expression RNA seq data.

## Materials and Methods

### TCGA Datasets

The TCGA gene expression RNAseq data (IlluminaHiSeq: log_2_-normalized_count+1) was downloaded from Xena browser (https://xenabrowser.net/datapages/) for three renal cancer types including clear cell carcinoma (KIRC−533 tumor, 72 normal), papillary carcinoma (KIRP−290 tumor, 32 normal), and chromophobe carcinoma (KICH−66 tumor, 25 normal) (26). Statistical analyses were performed to evaluate the expression levels of 32 STAT3-regulated genes in these RCC subtypes.

### Statistical Analyses

All statistical analyses were performed using the R language and environment for statistical computing (R version 3.2.2; R Foundation for Statistical Computing; www.r-project.org). The normalized counts were log2 transformed prior to all statistical analyses to achieve normal distribution. The potential differences in the gene expression between cancer patients and adjacent normal were initially examined using a *t*-test and Bonferroni correction (*p* < 0.0016) was used to adjust *p*-values for multiple testing. Boxplots were created to visualize the distribution of gene expression in cancer patients and adjacent normal. The biomarker potential of individual genes, which refers to the gene's diagnostic power to differentiate cancer patients from respective controls, was assessed using the area under the curve (AUC) of the receiver operating characteristic (ROC) curves.

## Results

**Figure 2:**
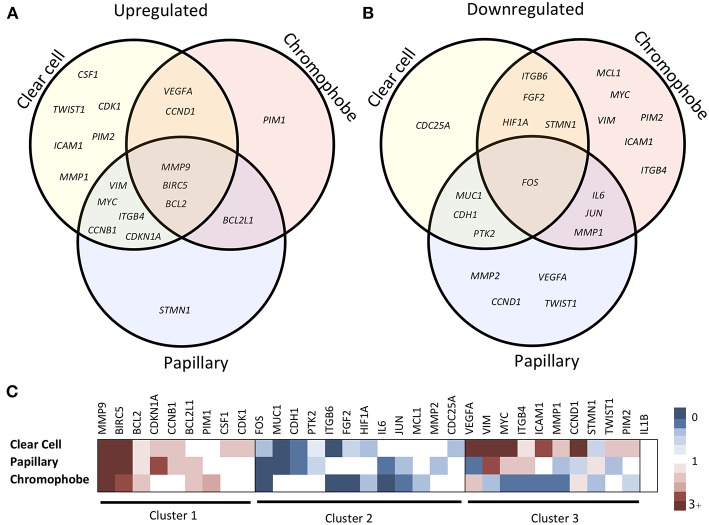
Comparison of STAT3-regulated gene expression changes in three RCC subtypes. Venn diagram showing significantly upregulated **(A)** and downregulated **(B)** genes across clear cell, papillary and chromophobe renal cancer subtypes. Heatmap **(C)** shows gene expression fold change values in tumor as compared to unmatched adjacent normal. Red color represents increased expression, blue represents decreased expression and white represents no significant change.

The expression fold change values (tumor vs. unmatched adjacent normal) for selected 32 STAT3-regulated genes in 3 different renal cancer subtypes are presented in Table 1. Comparisons of significantly upregulated and downregulated genes in the three cancer subtypes relative to normal tissue are shown using Venn diagrams (Figures 2A,B). Interestingly, using significance level α = 0.0016, three genes including *MMP9, BIRC5*, and *BCL2* were significantly upregulated whereas, only one gene (*FOS)* was significantly downregulated in all three subtypes. Expression of several genes varied substantially between the cancer subtypes suggesting significant differences in STAT3 pathway activation in these three renal cancer subtypes.

**Table 1:** STAT3-regulated gene expression fold change across RCC subtypes.

**Gene**	**Descriptions**	**Clear cell (KIRC)**		**Papillary (KIRP)**		**Chromophobe (KICH)**	
		**FC**	***P*-value**	**FC**	***P*-value**	**FC**	***P*-value**
**UPREGULATED**							
*MMP9*	Matrix metallopeptidase 9	9.90	6.4 × 10^−27^	3.77	1.4 × 10^−05^	3.24	0.0006
*BIRC5*	Baculoviral IAP repeat containing 5	7.42	4.1 × 10^−28^	6.95	3.6 × 10^−15^	2.89	7.4 × 10^−05^
*BCL2*	BCL2, apoptosis regulator	1.48	1.0 × 10^−24^	1.34	2.2 × 10^−06^	1.91	8.7 × 10^−09^
*CDKN1A*	Cyclin dependent kinase inhibitor 1A	1.93	6.6 × 10^−10^	3.00	1.5 × 10^−09^	0.80	0.3020
*BCL2L1*	BCL2 like 1	1.05	0.0216	1.53	3.5 × 10^−20^	1.78	1.5 × 10^−14^
*CCNB1*	cyclin B1	1.69	8.6 × 10^−19^	1.61	1.2 × 10^−12^	1.48	0.0023
*PIM1*	Pim-1 proto-oncogene, serine/threonine kinase	1.04	0.4540	1.12	0.1690	2.16	4.7 × 10^−08^
*CSF1*	Colony stimulating factor 1	1.80	2.9 × 10^−22^	0.86	0.0445	0.65	0.0020
*CDK1*	Cyclin dependent kinase 1	1.62	1.0 × 10^−10^	1.35	0.0018	0.91	0.4490
**DOWNREGULATED**							
*FOS*	Fos proto-oncogene, AP-1 transcription factor subunit	−1.96	2.8 × 10^−07^	−5.56	2.7 × 10^−14^	−7.14	1.6 × 10^−11^
*ITGB6*	Integrin subunit beta 6	−9.09	2.0 × 10^−35^	−1.45	0.0577	−7.14	7.0 × 10^−13^
*IL6*	Interleukin 6	−1.22	0.2310	−4.76	1.0 × 10^−07^	−11.11	3.7 × 10^−09^
*MUC1*	Mucin 1, cell surface associated	−5.00	3.2 × 10^−57^	−5.56	1.3 × 10^−23^	−1.47	0.0081
*CDH1*	Cadherin 1	−3.57	3.8 × 10^−44^	−4.17	2.1 × 10^−33^	1.49	0.0120
*FGF2*	Fibroblast growth factor 2	−1.56	7.1 × 10^−09^	−1.09	0.4290	−5.00	2.2 × 10^−14^
*JUN*	Jun proto-oncogene, AP-1 transcription factor subunit	1.18	0.0588	−1.72	1.3 × 10^−06^	−2.70	5.8 × 10^−07^
*HIF1A*	Hypoxia inducible factor 1 subunit alpha	−2.27	1.48 × 10^−21^	−1.18	0.0668	−1.69	7.68 × 10^−06^
*PTK2*	Protein tyrosine kinase 2	−1.08	2.6 × 10^−05^	−1.39	6.5 × 10^−23^	−1.08	0.1900
*MCL1*	MCL1, BCL2 family apoptosis regulator	1.11	0.0811	−1.05	0.5350	−2.38	1.0 × 10^−07^
*MMP2*	Matrix metallopeptidase 2	1.07	0.4350	−2.27	1.6 × 10^−09^	−1.43	0.1080
*CDC25A*	Cell division cycle 25A	−1.67	1.03 × 10^−12^	1.17	0.0087	1.37	0.0051
**MIXED**							
*VEGFA*	Vascular endothelial growth factor A	9.60	2.2 × 10^−64^	−2.78	2.1 × 10^−18^	1.81	5.8 × 10^−06^
*VIM*	Vimentin	6.10	9.9 × 10^−50^	2.94	1.2 × 10^−17^	−2.13	6.9 × 10^−06^
*ICAM1*	Intercellular adhesion molecule 1	2.90	1.63 × 10^−27^	1.34	0.0047	−3.45	1.17 × 10^−10^
*MYC*	MYC proto-oncogene, bHLH transcription factor	3.35	2.0 × 10^−19^	1.90	5.4 × 10^−05^	−2.94	5.0 × 10^−06^
*MMP1*	Matrix metallopeptidase 1	1.67	0.0010	−2.33	6.5 × 10^−06^	−3.33	5.1 × 10^−05^
*ITGB4*	Integrin subunit beta 4	1.27	0.0005	1.98	3.4 × 10^−08^	−2.70	1.7 × 10^−09^
*STMN1*	Stathmin 1	−1.28	7.2 × 10^−08^	1.26	0.0006	−1.85	3.4 × 10^−10^
*CCND1*	Cyclin D1	4.31	6.6 × 10^−83^	−1.39	4.2 × 10^−05^	1.36	0.0002
*TWIST1*	Twist family bHLH transcription factor 1	1.78	5.96 × 10^−09^	−2.08	3.34 × 10^−07^	−1.25	0.3640
*PIM2*	Pim-2 proto-oncogene, serine/threonine kinase	1.87	7.4 × 10^−25^	1.03	0.6970	−1.52	0.0007
**NO CHANGE**							
*IL1B*	Interleukin 1 beta	1.40	0.0161	0.91	0.5770	0.44	0.0018

### STAT3-Regulated Gene Expression Changes in Clear Cell RCC

A total of 16 genes were significantly upregulated and 9 genes were significantly downregulated in clear cell carcinoma. The genes with more than 2-fold upregulation include *MMP9* (9.90-fold), *VEGFA* (9.60-fold), *BIRC5* (7.42-fold), *VIM* (6.10-fold), *CCND1* (4.31-fold), *MYC* (3.35-fold) and *ICAM1* (2.90-fold). Six genes including *ICAM1* (2.90-fold)*, PIM2* (1.87-fold), *CSF1* (1.80-fold)*, MMP1* (1.67-fold)*, TWIST1* (1.78-fold), and *CDK1* (1.62-fold) were upregulated only in clear cell, but not in papillary or chromophobe. Five most downregulated genes in clear cell carcinoma include, *ITGB6* (−9.09-fold), *MUC1* (−5.00-fold), *CDH1* (−3.57-fold), *HIF1A* (−2.27-fold) and *FOS* (−1.96-fold). Among the downregulated genes *CDC25A* (−1.67-fold) was downregulated only in clear cell carcinoma but not in other two subtypes.

### STAT3-Regulated Gene Expression Changes in Papillary RCC

A total of 21 genes were significantly changed (10 upregulated and 11 downregulated) in papillary renal carcinoma. Genes with greatest upregulation (>2-fold) include *BIRC5* (6.95-fold), *MMP9* (3.77-fold), *VIM* (2.94-fold), and *CDKN1A* (3.00-fold). *STMN1* gene was slightly upregulated in papillary (1.26-fold), whereas it was downregulated in clear cell and chromophobe. Genes with more than 2-fold downregulation in papillary include, *FOS* (−5.56-fold), *IL6* (−4.76-fold), *MUC1* (−5.56-fold), *CDH1* (−4.17-fold), *MMP2* (−2.27-fold), *VEGFA* (−2.78-fold), *MMP1* (−2.33-fold), and *TWIST1* (−2.08-fold). Of note, among the downregulated genes *MMP2, VEGFA, CCND1*, and *TWIST1* were downregulated only in papillary carcinoma but were either unchanged or upregulated in other two subtypes.

### STAT3-Regulated Gene Expression Changes in Chromophobe RCC

In chromophobe we found significant alterations in the expression of 21 genes (7 upregulated and 14 downregulated). Three genes with more than 2-fold upregulation are *BIRC5* (2.89-fold), *MMP9* (3.24-fold), and *PIM1* (2.16-fold). *PIM1* gene was only upregulated in chromophobe and it was unchanged in clear cell and papillary subtypes. Almost half of the STAT3 regulated genes were significantly downregulated in chromophobe. Genes with more than 2-fold downregulation in chromophobe include, *FOS* (−7.14-fold), *ITGB6* (−7.14-fold), *IL6* (−11.11-fold), *FGF2* (−5.00-fold), *JUN* (−2.70-fold), *MCL1* (−2.38-fold), *VIM* (−2.13-fold), *ICAM1* (−3.45-fold), *MYC* (−2.94-fold), *MMP1* (−3.33-fold), and *ITGB4* (−2.70-fold). Among these genes *MYC, VIM, ICAM1*, and *ITGB4* were downregulated only in chromophobe but upregulated in other two subtypes.

### Gene Expression Similarities and Differences Between Three Renal Cancer Subtypes

**Figure 3:**
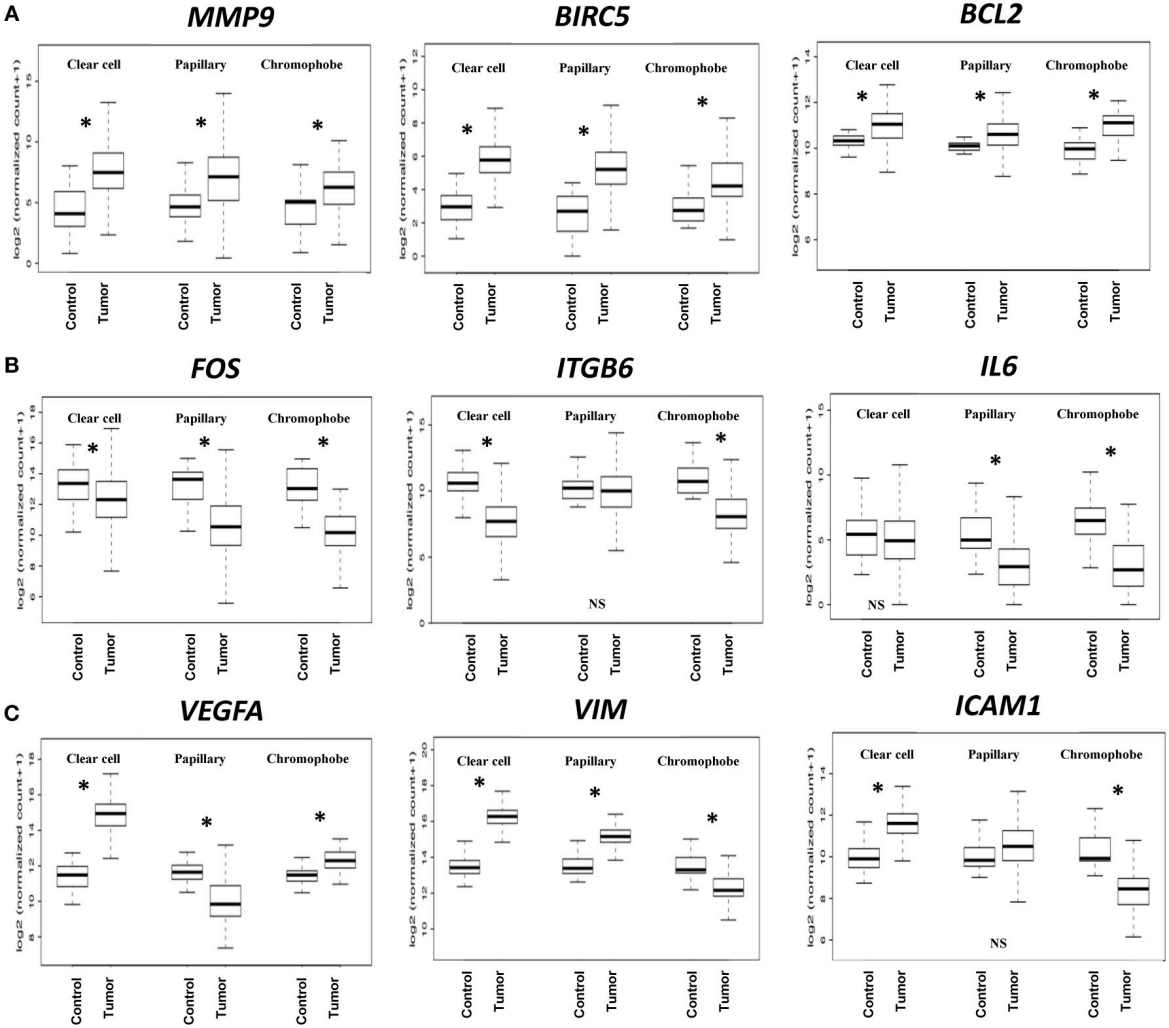
Boxplots of relative gene expression across RCC subtypes. The boxplots represent the distribution of expression in tumor and control samples. **(A)** *MMP9, BIRC5*, and *BCL2* are significantly upregulated in all three subtypes; **(B)** *FOS, ITGB6*, and *IL6* genes are either downregulated or not changed; **(C)** *VEGFA, VIM*, and *ICAM1* have variable expression across subtypes. ^*^p < 0.0016, NS = not significant.

Based on the expression patterns, we divided all 32 genes into four groups (up, down, mixed, no change) (Figure 2C). A cluster of 9 genes including *MMP9, BIRC5, BCL2, CDKN1A, BCL2L1, CCNB1, PIM1, CSF1*, and *CDK1* was either upregulated or unchanged but was not downregulated in any subtype. On the other hand, another cluster of 12 genes including *FOS, ITGB6, IL6, MUC1, CDH1, FGF2, JUN, HIF1A, PTK2, MCL1, MMP2*, and *CDC25A* was either downregulated or unchanged but was not upregulated in any subtype. Another group of 10 genes including *VEGFA, VIM, ICAM1, MYC, MMP1, ITGB4, STMN1, CCND1, TWIST1*, and *PIM2* had considerable variation between subtypes. For example, *VEGFA* was upregulated in clear cell (9.60-fold) and chromophobe (1.81-fold) but downregulated in papillary (−2.78-fold) (Figure 3). *VIM* was upregulated in clear cell (6.10-fold) and papillary (2.94-fold) but downregulated in chromophobe (−2.13-fold). *ICAM1* was upregulated in clear cell (2.90-fold), downregulated in chromophobe (−3.45-fold), and not significantly changed in papillary. Similarly, remaining genes of this cluster had a mixed expression pattern as shown in Figure 2C. Only the expression of *IL1B* was unchanged in all three subtypes.

### Evaluation of Biomarker Potential of the STAT3-Regulated Genes

**Figure 4:**
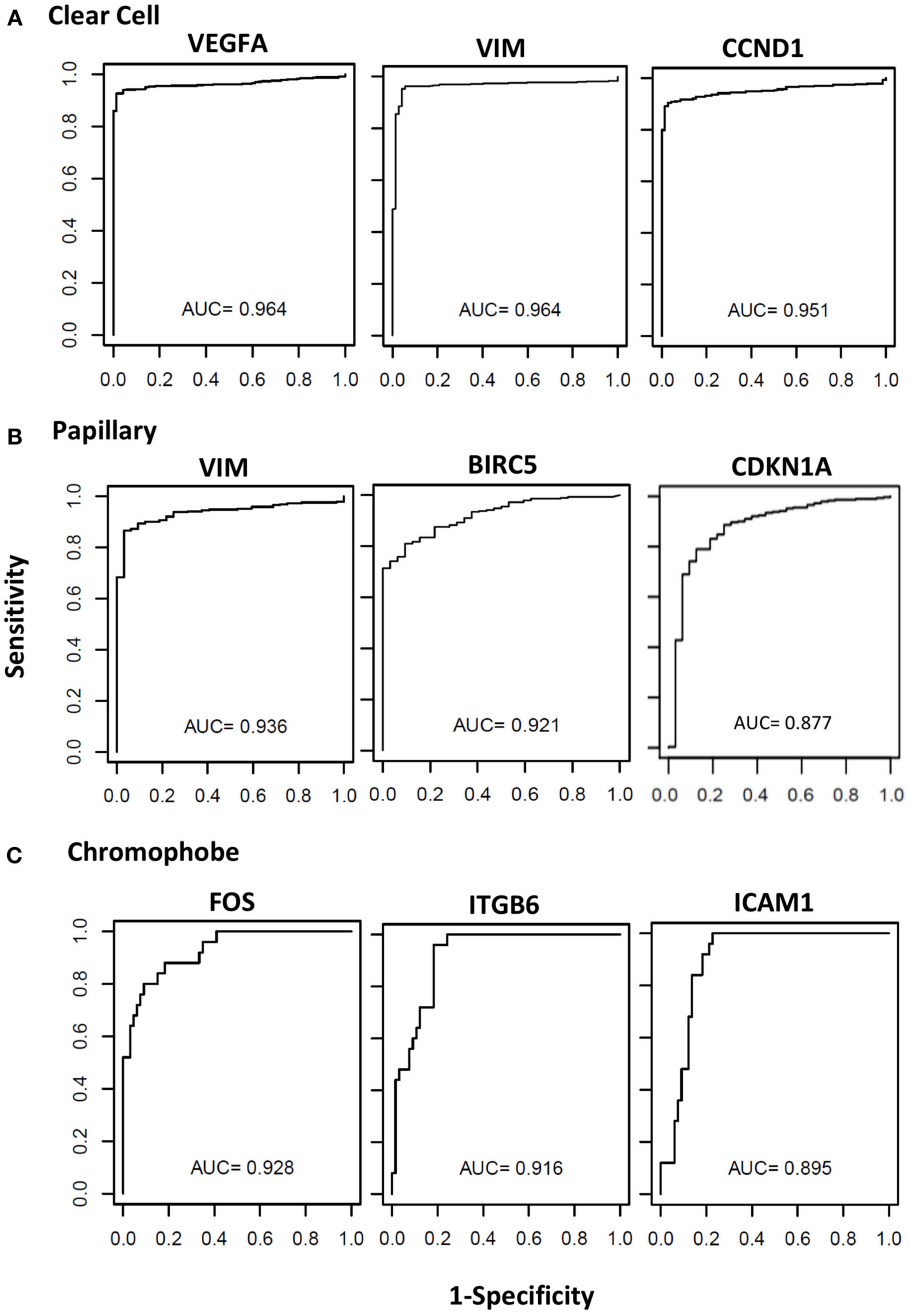
Receiver operating curves (ROC) of STAT3-regualed genes. The diagnostic power of individual genes to differentiate cancer patients and respective controls was assessed using the area under the curve (AUC) of the receiver operating characteristic (ROC) curves. The ROC curves and AUC values of top three performing genes in clear cell **(A)**, papillary **(B)**, and chromophobe **(C)** are shown.

The biomarker potential of the genes was evaluated using the Receiver Operator Characteristic (ROC) analyses using cases and controls. The Area under the Curve (AUC) values for all the genes in the three cancer types are presented in Supplemental Table 1. The ROC curves for some representative genes with the highest AUC values for each renal cancer type are presented in Figure 4. In clear cell, eight genes had an excellent biomarker potential with AUC values > 0.9 (*VEGFA*: 0.964, *VIM*: 0.964, *CCND1*: 0.951, *ITGB6*: 0.935, *BIRC5*: 0.934, *MUC1*: 0.924, *CDH1*: 0.923, and *ICAM1*: 0.902). Two genes had AUC values > 0.9 in both papillary (*VIM*: 0.936, *BIRC5*: 0.921) and chromophobe (*FOS*: 0.928, *ITGB6*: 0.916).

## Discussion

Renal cell carcinoma is the ninth most common malignancy worldwide, and as many as 25% of RCC patients have metastatic disease at diagnosis. Despite significant improvements in survival over the past decade, patients with stage III and stage IV RCC have 5-year survival rates of only 53 and 8%, respectively (27). Approximately 70% of RCC tumors have clear cell histology, and while it is well-established that loss of the von Hippel-Lindau (*VHL*) tumor suppressor gene is the common mechanism of tumorigenesis in familial and sporadic clear cell tumors, many other clinically significant gene mutations have been identified and these tumors show significant genetic variability (28, 29). Papillary tumors, which are classified as type I or type II based on tumor histology, account for 10% of RCC tumors, and while familial cases are linked to *c-MET* mutations the cause of sporadic cases remains unclear (28, 29). Chromophobe tumors account for < 5% of RCC tumors and typically show whole chromosome deletions, though the impact of these losses has not been fully characterized; mutations in *PTEN* and *TP53* have been identified, but they are only present in a minority of cases (28, 29). The majority of both basic science and clinical studies focus mostly on clear cell RCC and exclude other subtypes. Also, despite clear differences in molecular pathogenesis, current treatment guidelines for all types of RCC are the same.

The transcription factor STAT3 is known to be important for renal development and tubulogenesis, and high levels of STAT3 activation have been observed during periods of active kidney growth in newborn mice (30–32). While STAT3 is highly expressed in adult kidney tissue, its activity under normal, healthy conditions is extremely low (33). Because of this high expression, however, STAT3 can be activated very quickly in response to stimuli. In a model of acute kidney injury induced by HgCl_2_, STAT3 activation in renal tissue has been linked to interleukin-6 (IL-6) signaling; because normal renal tissue does not express the IL-6 receptor, this activity has been linked to IL-6 trans-signaling, which involves signaling through a soluble form of the IL-6 receptor (34).

Additionally, it is well established that increased STAT3 activation correlates with both advanced metastatic disease and worse survival in RCC. Furthermore, the majority of this increased activity is due to overstimulation of STAT3-linked receptors by increased growth factor and cytokine signaling rather than constitutive activation (4). Multi-kinase inhibitors sunitinib and sorafenib target many of these receptors and have shown promise in both *in vitro* models and clinical trials (35, 36). A clinical trial of both of these drugs in papillary and chromophobe patients showed prolonged progression-free survival, though chromophobe patients showed much better response than papillary (37). Another analysis measuring STAT3 activation across RCC subtypes by detection of phosphorylated STAT3 in tissue microarray showed similar numbers of tumors with activated STAT3 in clear cell and papillary RCC (57–59%), while fewer chromophobe cases (33%) showed STAT3 activation; however, the small sample size and possibility of false negatives due to small tissue core size may limit the usefulness of these conclusions (1).

In the present study, we utilized TCGA gene expression dataset to compare expression of 32 genes that are regulated by STAT3 to both evaluate the STAT3 activation across RCC subtypes and to further analyze the downstream effects of this activation. Overall, our analysis indicates that STAT3 activation plays a pivotal role in clear cell RCC, as 16 of the 32 genes evaluated were upregulated in this subtype compared to only 10 in papillary and 7 in chromophobe. Clear cell and papillary showed the most similarities in gene expression, with 11 genes showing similar expression patterns. For confirmation, we repeated this analysis using Gene Expression Omnibus (GEO) dataset GSE6344 (38), which measured gene expression in clear cell RCC tumors and matched normal tissue. Out of the 16 significantly upregulated genes in our analysis of clear cell RCC, we could confirm the increased expression of 8 genes in the GSE6344 dataset, including *VEGFA, BIRC5, VIM, CCND1, MYC, CDKN1A, CDK1*, and *BCL2*. Among the nine downregulated genes in clear cell carcinoma, three gene including *STMN1, HIF1A*, and *MUC1* could be confirmed in this new dataset.

We identified a cluster of 9 genes that were either upregulated or unchanged but not downregulated in any subtype. Three of these genes, including matrix metalloproteinase 9 (*MMP9*), survivin (*BIRC5*), and B cell lymphoma 2 (*BCL2*), were significantly upregulated in all three subtypes. The roles of these genes in RCC have already been well established (39–41). *CDKN1A*, which encodes the protein p21 that inhibits cell proliferation, was upregulated in clear cell and papillary, and its expression is associated with decreased proliferation in RCC cell lines (42). *BCL2L1*, of which the major protein product is Bcl-xl, is a well-known inhibitor of apoptosis and was upregulated in papillary and chromophobe RCC. Interestingly, its overexpression is linked to gain or amplification of chromosome 20q, which has been reported in RCC, and STAT3 inhibition has been shown to decrease *BCL2L1* expression in RCC cell lines (36, 43, 44). *CCNB1* (cyclin B1) was upregulated in clear cell and papillary RCC in our analysis, and its overexpression has been linked to poor survival in all three subtypes of RCC (45). *PIM1* was only overexpressed in chromophobe RCC in our analysis, but its overexpression has been reported in many cancers, including RCC, and it has been shown to be a promising therapeutic target using RCC cell lines (46). *CDK1* (cyclin-dependent kinase 1) was upregulated in clear cell in our analysis and has shown to be associated with worse survival and recurrence in RCC (47). *CSF1* (macrophage colony-stimulating factor) was overexpressed in clear cell RCC in our analysis. Sunitinib, which inhibits many receptors related to STAT3 including the CSF-1 receptor, has been shown to decrease myeloid-derived suppressor cell (MDSC) levels in RCC patients in clinical trial, though it is difficult to determine which specific receptor may be responsible for this effect (48). Interestingly, the small molecule inhibitor specific to the CSF-1 receptor GW2580 showed promise in decreasing MDSC recruitment and function in an *in vivo* tumor model, and exploration into its effectiveness in clear cell RCC should be further explored (49).

Among another cluster of 10 genes that showed variable expression changes across subtypes (*VEGFA, VIM, ICAM1, MYC, MMP1, ITGB4, STMN1, CCND1, TWIST1, PIM2*), 9 were upregulated in clear cell (Figure 2). In the papillary subtype, 4 of these genes were upregulated and another 4 were downregulated, whereas in chromophobe 7 of these were downregulated. *VEGFA* was upregulated in clear cell and chromophobe, but was downregulated in papillary RCC. The role of VEGF in clear cell RCC is well known, and bevacizumab, a VEGF inhibitor, is a well-established therapy for use in combination with erlotinib, a receptor tyrosine kinase inhibitor, to treat advanced metastatic clear cell RCC (50). *VIM* (vimentin) was upregulated in both clear cell and papillary, but downregulated in chromophobe. Vimentin has been established as a histological marker for distinguishing clear cell from chromophobe RCC, as chromophobe is considered to be negative for vimentin expression as only 2% express vimentin (51). *ICAM1* (intracellular adhesion molecule 1) was upregulated in clear cell, unchanged in papillary, and downregulated in chromophobe; while ICAM1 expression is linked to increased leukocyte infiltration of RCC tumors, much is still unknown about endothelial activation and RCC disease progression (52, 53). *MYC* is known to be upregulated in clear cell and papillary RCC, which is consistent with our analysis (54–56). *MYC* was downregulated in chromophobe RCC, and to our knowledge *MYC* expression in chromophobe RCC has not been previously evaluated. *MMP1* was upregulated in clear cell, and downregulated in both papillary and chromophobe. Interestingly, a polymorphism causing increased expression of MMP1 has been linked to increased risk of many tumors, including RCC, but the risk is only seen in males, suggesting MMP1 regulation may be sex-dependent (57). *ITGB4* (integrin beta 4) was upregulated in both clear cell and papillary, and downregulated in chromophobe. While increased *ITGB4* overexpression has been correlated with metastasis in RCC, to our knowledge, the differential expression of *ITGB4* across RCC subtypes has never before been reported (58). Interestingly, *STMN1* was the only gene to be upregulated in papillary, but downregulated in both clear cell and chromophobe. In an immunohistochemistry analysis of RCC tumors, all three subtypes stained positively for stathmin, but papillary tumors showed the highest percentage of strongly positive staining (59). *CCND1* (cyclin D1) was upregulated in clear cell and chromophobe, but was downregulated in papillary; overexpression in clear cell RCC is linked to loss of the VHL gene (60). *TWIST1* (twist-related protein 1) was upregulated in clear cell, downregulated in papillary, and unchanged in chromophobe. *TWIST1* is a transcription factor and its cellular location plays an important role in its activity; a recent study showed that a high level of cytoplasmic *TWIST1* is an indicator of poor prognosis (61). *PIM2* (serine/threonine-protein kinase Pim-2) was upregulated in clear cell, downregulated in chromophobe, and unchanged in papillary. In clear cell RCC, increased *PIM2* expression is correlated with more advanced disease and metastasis (62).

We also identified a cluster of 12 genes which was downregulated or unchanged, but not upregulated in any subtype. *FOS* (c-Fos) was the only gene that was significantly downregulated in all three RCC subtypes in our analysis; this protein dimerizes with c-Jun to form the transcription factor AP-1, which is involved in cell proliferation. Of note, c-Fos activation is linked to *VHL* inactivation, and elevated c-FOS expression is correlated with worse survival (63). *JUN* (c-Jun) was unchanged in clear cell, but downregulated in both papillary and chromophobe. *ITGB6* (integrin beta 6), *FGF2* (basic fibroblast growth factor, FGF-β) and *HIF1A* were strongly downregulated in clear cell and chromophobe, but unchanged in papillary. While *ITGB6* has been described in subclinical inflammation in normal renal tissue, but to our knowledge it has not been evaluated in RCC (64). FGF-β plays a role in RCC tumor growth, its expression is not correlated with clinical outcomes (65). Also, loss of normal *HIF1A* regulation, which is normally degraded in normoxic conditions, is linked to loss of the *VHL* gene in RCC (66). *IL6* (interleukin-6) was unchanged in clear cell, but downregulated in both papillary and chromophobe in our analysis. This contradicts published data showing that RCC tumors express much higher levels of *IL6* than normal renal tissue and RCC cell lines utilize IL-6 as an autocrine growth factor (67). *MUC1* (mucin 1), *CDH1* (cadherin-1, E-cadherin), and *PTK2* (protein tyrosine kinase 2 or focal adhesion kinase [FAK]) were downregulated in clear cell and papillary, but unchanged in chromophobe. *MUC1* has previously been described as a prognostic marker in RCC (68), whereas loss of E-cadherin is associated with metastasis and poor prognosis in RCC (69). Though FAK levels are increased in many cancers, but seem to have not been implicated in RCC tumor formation (70). *MCL1* (Mcl-1, Bcl2L3), an anti-apoptotic gene in the Bcl2 family, was downregulated in chromophobe and unchanged in clear cell and papillary. In RCC, Mcl-1 expression is linked to TNF-alpha-related apoptosis-inducing ligand (TRAIL) resistance, and sorafenib has been shown to reduce both expression of Mcl-1 and TRAIL resistance (71). *MMP2* was downregulated in papillary RCC in our analysis, but interestingly it has been shown that like MMP9, increased expression of MMP2 in RCC is an indicator of poor prognosis across subtypes (39). *CDC25A* was only downregulated in clear cell RCC; *CDC25A* expression has been shown to be inhibited by sunitinib in RCC (36).

Nine STAT3-regulated genes (*VEGFA, VIM, VVND1, ITGB6, BIRC5, MUC1, CDH1, ICAM1*, and *FOS*) were identified as potential biomarkers to distinguish tumor from normal renal tissue by ROC analyses, with AUC values > 0.9 in at least one RCC subtype. *VEGFA* was identified as a candidate biomarker in clear cell, and as previously mentioned its role in clear cell RCC is well-established (50). *VIM* was identified as a candidate biomarker for both clear cell and papillary, and was significantly upregulated in both subtypes in our analysis. Vimentin has already been established as an immunohistochemistry marker to distinguish clear cell and papillary from both normal renal tissue and other renal cancer types (72). Additionally, vimentin expression has been correlated with poor survival in RCC patients (73), though this study did not differentiate between tumor subtypes. *CCND1* showed biomarker potential and high expression in clear cell in our analysis, and though high cyclin D1 has been correlated with better prognosis in clear cell, expression alone as measured by immunohistochemistry was not an independent prognostic factor (74). *ITGB6* was identified as a candidate biomarker in clear cell and chromophobe, and it was significantly downregulated in both subtypes. As previously noted, to our knowledge this gene has not been evaluated in RCC and both its role in RCC biology and biomarker potential should be further explored. *BIRC5* was strongly upregulated in all three subtypes, but was only identified as a candidate biomarker in clear cell and papillary. Increased expression of survivin is well-characterized in RCC, and though high survivin expression has been correlated with increased tumor aggressiveness and poor prognosis in RCC overall, the prognostic value of survivin expression has not been explored in individual subtypes (75, 76). *MUC1* showed biomarker potential in clear cell, in which it was strongly downregulated. Similar expression patterns have been reported, though differences in cytoplasmic and membranous expression were observed in benign, malignant, and metastatic RCC tissues, and its role as a biomarker for clear cell RCC should be evaluated further (77). *CDH1* was identified as a candidate biomarker in clear cell, in which it was downregulated, and as previously mentioned the loss of *CHD1* has been associated with metastasis and poor prognosis (69). *ICAM1* showed was upregulated in clear cell, and its biomarker potential in this subtype should be further explored. Finally, *FOS* was downregulated in all subtypes but only showed biomarker potential in chromophobe, where it had the highest AUC value in this subtype. As previously mentioned elevated c-FOS expression has been correlated with worse survival, but this study was limited to clear cell cases, and the role of *FOS* in chromophobe RCC should be evaluated (63).

In conclusion, STAT3 signaling is linked to cancer proliferation, survival, invasion, angiogenesis, metastasis, and inflammation, and has been shown to be an effective therapeutic target in RCC treatment. Overall our results suggest that STAT3 signaling differs between clear cell, papillary, and chromophobe RCC, and that it plays a more significant role in clear cell than in the other subtypes. Of the genes evaluated, we found specific genes upregulated or downregulated in RCC subtypes, and their roles in the pathogenesis of RCC should be further explored, including as potential therapeutic targets. The unmet need is to develop therapies/drugs targeting genes associated with STAT3 pathway in the renal cancer subtypes.

## Data Availability

The datasets analyzed for this study can be found in The Cancer Genome Atlas (https://cancergenome.nih.gov).

## Author Contributions

RR, AS, and SS wrote the manuscript and created figures and tables. RR, AS, SB, TL, and SK performed data analysis. RR, AS, SS, SH, and NP contributed to data interpretation and made revisions to the manuscript, tables, and figures.

### Conflict of Interest Statement

The authors declare that the research was conducted in the absence of any commercial or financial relationships that could be construed as a potential conflict of interest.
