# Supplementary material for: Comparative STAT3-Regulated Gene Expression Profile in Renal Cell Carcinoma Subtypes
Source: Front Oncol. 2019 Feb 26;9:72. doi: 10.3389/fonc.2019.00072 (PMC6399114; doi:10.3389/fonc.2019.00072)
Supplement: Supplementary file 1 [file Table_1.DOCX]

**Supplemental Table 1. Area under the curve (AUC) values across RCC subtypes**

| **Gene** | **KIRC AUC** | **KIRP AUC** | **KICH AUC** |
| --- | --- | --- | --- |
| VIM | 0.964 | 0.936 | 0.831 |
| BIRC5 | 0.934 | 0.921 | 0.781 |
| ITGB6 | 0.935 | 0.553 | 0.916 |
| CCND1 | 0.951 | 0.656 | 0.800 |
| ICAM1 | 0.902 | 0.654 | 0.895 |
| FOS | 0.676 | 0.866 | 0.928 |
| CDH1 | 0.923 | 0.872 | 0.796 |
| VEGFA | 0.964 | 0.845 | 0.846 |
| MUC1 | 0.924 | 0.840 | 0.621 |
| CDKN1A | 0.768 | 0.877 | 0.544 |
| BCL2L1 | 0.538 | 0.868 | 0.868 |
| PTK2 | 0.594 | 0.853 | 0.552 |
| IL6 | 0.552 | 0.802 | 0.874 |
| MMP2 | 0.525 | 0.785 | 0.630 |
| ITGB4 | 0.614 | 0.777 | 0.878 |
| CCNB1 | 0.815 | 0.768 | 0.663 |
| TWIST1 | 0.704 | 0.765 | 0.695 |
| JUN | 0.573 | 0.757 | 0.828 |
| BCL2 | 0.780 | 0.750 | 0.853 |
| MMP9 | 0.882 | 0.724 | 0.733 |
| MMP1 | 0.619 | 0.719 | 0.748 |
| MYC | 0.868 | 0.709 | 0.823 |
| STMN1 | 0.685 | 0.680 | 0.881 |
| CDC25A | 0.768 | 0.604 | 0.672 |
| CDK1 | 0.752 | 0.588 | 0.607 |
| CSF1 | 0.835 | 0.582 | 0.698 |
| HIF1A | 0.888 | 0.562 | 0.792 |
| PIM1 | 0.523 | 0.552 | 0.861 |
| IL1B | 0.611 | 0.516 | 0.685 |
| MCL1 | 0.596 | 0.516 | 0.885 |
| PIM2 | 0.821 | 0.500 | 0.730 |
| FGF2 | 0.670 | 0.461 | 0.855 |
